# 3D-assisted ERCP enhances cannulation efficiency in malignant biliary obstruction: a prospective two-center study

**DOI:** 10.1097/JS9.0000000000003302

**Published:** 2025-10-17

**Authors:** Mengke Fan, Guochen Shang, Songxiang Liu, Tianhang Li, Dongliang Li, Zhen Ding, Zhewei Ye, Rong Lin

**Affiliations:** a Department of Gastroenterology, Union Hospital, Tongji Medical College, Huazhong University of Science and Technology, Wuhan, China; b Department of Gastroenterology, The First Affiliated Hospital of Shihezi University, Shihezi, China; c Department of Orthopedics, Union Hospital, Tongji Medical College, Huazhong University of Science and Technology, Wuhan, China; d Intelligent Medical Laboratory, Union Hospital, Tongji Medical College, Huazhong University of Science and Technology, Wuhan, China; e Nanyang Technological University, Singapore; f Department of General Surgery, Lu’An Affiliated Hospital of Anhui Medical University, Lu’An, Anhui, China; g Endoscopy Center, The First Affiliated Hospital of Sun Yat-sen University, Guangzhou, Guangdong Province, China

**Keywords:** biliary drainage, endoscopic retrograde cholangiopancreatography, malignant biliary obstruction, pancreatic cancer, three-dimensional reconstruction

## Abstract

**Background and Aims:**

Endoscopic retrograde cholangiopancreatography (ERCP)-guided biliary drainage is the standard treatment for malignant biliary obstruction in patients who are not candidates for curative surgery. However, ERCP can be more challenging in patients with pancreatic cancer complicated by obstructive jaundice, often leading to prolonged cannulation times. This study aimed to evaluate the efficacy of three-dimensional (3D) reconstruction-assisted ERCP in treating patients with pancreatic cancer complicated by malignant biliary obstruction.

**Methods:**

We performed a two-center prospective study including 72 patients diagnosed with pancreatic cancer complicated by obstructive jaundice. The 36 patients who underwent 3D reconstruction before ERCP constituted the intervention group, while the remaining patients, who received conventional ERCP without 3D assistance, served as the control group. Outcomes evaluated included technical success, procedure duration, X-ray exposure time, clinical success (defined as a ≥50% reduction in serum bilirubin within 5 days), and adverse events.

**Results:**

3D models were successfully constructed for patients in the intervention group and were used for both preoperative planning and intraoperative guidance during ERCP. Compared to conventional ERCP, 3D-assisted ERCP significantly reduced cannulation time (16 min [IQR: 13–30] vs. 13.5 min [IQR: 9–21], *P* = 0.036) and X-ray exposure time (40.5 s [IQR: 28–69] vs. 34.5 s [IQR: 24–45.5], *P* = 0.034) for both patients and physicians. No significant differences were observed between the two groups in terms of technical success rates (97.2% vs. 100%, *P* = 1), clinical success rates (77.8% vs. 83.3%, *P* = 0.77) or adverse event rates (19.44% vs. 13.89%, *P* = 0.75). Additionally, no procedure-related mortality occurred.

**Conclusions:**

3D-assisted ERCP significantly reduces the X-ray exposure time and shortens cannulation time for both patients and physicians, making it a valuable tool for treating malignant biliary obstruction.

**Highlights:**

This prospective two-center study demonstrates that 3D reconstruction-assisted ERCP can significantly reduce X-ray exposure time and overall procedure duration, thereby enhancing safety and efficiency in the management of malignant biliary obstruction.

**HIGHLIGHTS**

- Compared to conventional two-dimensional imaging modalities such as CT or MRI, three-dimensional (3D) reconstruction delivers superior anatomical detail and more intuitive spatial visualization of the biliary and pancreatic ductal systems.
- 3D reconstruction-assisted ERCP significantly shortens cannulation time and reduces X-ray exposure during treatment of malignant biliary obstruction secondary to pancreatic cancer, thereby improving both procedural safety and efficiency.
- This approach holds considerable potential for enhancing clinical outcomes and procedural success rates in therapeutic endoscopy, particularly in technically complex ERCP cases.

## Introduction

Pancreatic cancer is one of the most common malignant tumor of the digestive system. The data showed that the overall 5-year survival rate for all stages of pancreatic cancer is only 5%^[1,2]^. Approximately 70% of patients with pancreatic cancer are diagnosed with malignant biliary obstruction^[3,4]^, which may lead to liver dysfunction, biliary cirrhosis and an increased incidence of cholangitis.

With the continuous improvement of endoscopic technology, endoscopic retrograde cholangiopancreatography (ERCP) has become an essential tool for the treatment of patients with biliary and pancreatic diseases^[5]^. Endoscopic biliary stent placement is a commonly used palliative treatment to relieve jaundice, offering lower costs, fewer procedure-related complications, improved quality of life, and shorter hospital stays^[3]^. However, surgeons are at high risk and require sufficient surgical experience and skills^[6]^. Besides, due to tumor invasion, the positional relationship between the bile duct and obstruction patterns becomes complex, making selective biliary cannulation technically challenging and sometimes unsuccessful^[7]^. The correct and precise selection of the target bile duct is crucial for successful selective biliary cannulation. Currently, target bile duct selection primarily relies on preoperative computed tomography (CT), magnetic resonance cholangiopancreatography (MRCP), and intraoperative cholangiography^[8]^. However, in cases of complex malignant biliary strictures, these modalities may be insufficient to clearly define the optimal cannulation path^[9]^. Cannulation of difficult bile ducts contributes to a higher incidence of adverse events and increased radiation exposure for both patients and endoscopists^[10,11]^. Therefore, there is an urgent need to develop new technologies that can improve the efficacy and safety of ERCP.

In recent years, the application of three-dimensional (3D) reconstruction technology has become increasingly extensive in various medical specialties. In surgery and interventional radiology, 3D reconstruction technology has been employed for preoperative planning, providing enhanced anatomical detail and improved spatial orientation^[12,13]^. Reports have indicated that these systems help shorten procedure times, improve the procedure success rate, and reduce complication risks. By comparing conventional two-dimensional (2D) imaging modalities such as CT or magnetic resonance imaging, 3D reconstruction provides more detailed anatomical information and a more intuitive spatial structure of the biliary tract and pancreatic duct. Its value in the surgical treatment of pancreatic cancer has been well recognized^[14,15]^. In addition, these models can be freely rotated, enlarged, or rendered semitransparent, helping determine the optimal target bile duct, guide selective cannulation. However, the integration of 3D reconstruction into ERCP remains in the early stages, with limited evidence supporting its clinical application. A retrospective study of 15 patients with hilar cholangiocarcinoma found that 3D reconstruction may assist in ERCP planning and improve procedural outcomes^[16]^. Another small retrospective study showed 3D reconstruction offered superior anatomical visualization compared to MRCP and could help improve endoscopic management in patients with malignant hilar stricture^[17]^. Despite these promising findings, both studies are constrained by retrospective designs and small sample sizes, and no prospective, multicenter evidence currently exists to validate the role of 3D reconstruction in ERCP.

Therefore, this two-center prospective study was conducted to evaluate the efficacy of 3D reconstruction in guiding ERCP cannulation for patients with pancreatic cancer complicated by malignant biliary obstruction.

## Material and methods

### Participants

This was a two-center prospective study conducted between December 2021 and February 2023. We enrolled 72 consecutive patients diagnosed with pancreatic cancer complicated by obstructive jaundice and treated with ERCP. Sample size estimation was conducted to detect a 10-second reduction in X-ray exposure time between the 3D-assisted and conventional ERCP groups, assuming a standard deviation of 15 seconds. With a two-sided alpha of 0.05 and a power of 80%, a minimum of 36 patients per group (72 total) was required. Patient eligibility for ERCP was determined by a multidisciplinary team based on clinical condition, imaging findings, and anatomical considerations. Only patients with normal gastrointestinal anatomy and malignant biliary obstruction caused by pancreatic cancer were included. Patients with duodenal obstruction, altered surgical anatomy, or contraindications to endoscopic intervention were excluded. The study’s inclusion criteria are outlined in Table 1. Patients were randomly assigned to either conventional ERCP group or 3D-assisted ERCP group. This case-control study has been reported in line with the STROCSS guidelines^[18,19]^.

**Table 1:** Selection criteria

Inclusion criteria		
	1. CT or MRI of the abdomen shows a pancreatic mass with a histological or cytological diagnosis of malignancy
	2. Serum bilirubin exceeds at least three times the upper limit of normal
	3. Cannot be treated surgically
	4. Provide informed consent
**Exclusion criteria**	
	1. Surgical alteration of the anatomy or inability to access the major duodenal papilla
	2. Cirrhosis with portal hypertension and/or ascites
	3. Cachexia, liver function is in a state of failure, bile drainage is no longer clinically relevant
	4. Advanced tumors with multiple metastases and ERCP procedures have a high impact on cardiopulmonary function and high risk
	5. Those who are allergic to iodine contrast agent or have severe coagulation disorders
	6. Prior ERCP attempts or biliary interventions

CT, computed tomography; ERCP, endoscopic retrograde cholangiopancreatography; MRI, magnetic resonance imaging.

### Data acquisition and 3D reconstruction

Abdominal enhanced CT scans were performed using a 128-slice spiral CT scanner (GE Corporation, Stamford, CT, United States) with a 0.625-mm slice interval. A high-pressure syringe was used to inject a contrast agent (Bayer AG, Leverkusen, Germany). The collected image data were then imported into a 3D visualization system (Mimics 17.0; Materialise Co., Leuven, Belgium) to reconstruct 3D models of the pancreatic tumor, bile ducts, and pancreatic ducts. The 3D models could be manipulated (magnified, reduced, rotated, or made transparent) for detailed visualization of the size, shape, and position of the lesions, as well as the diameters of the bile and pancreatic ducts. Modeling was completed by trained technicians and typically required approximately 30–60 minutes. The process was integrated into the preoperative workflow and did not cause delays in the clinical schedule. These models aided in preoperative planning and the selection of the optimal cannulation pathway.

### ERCP with biliary stent placement

**Figure 1.**
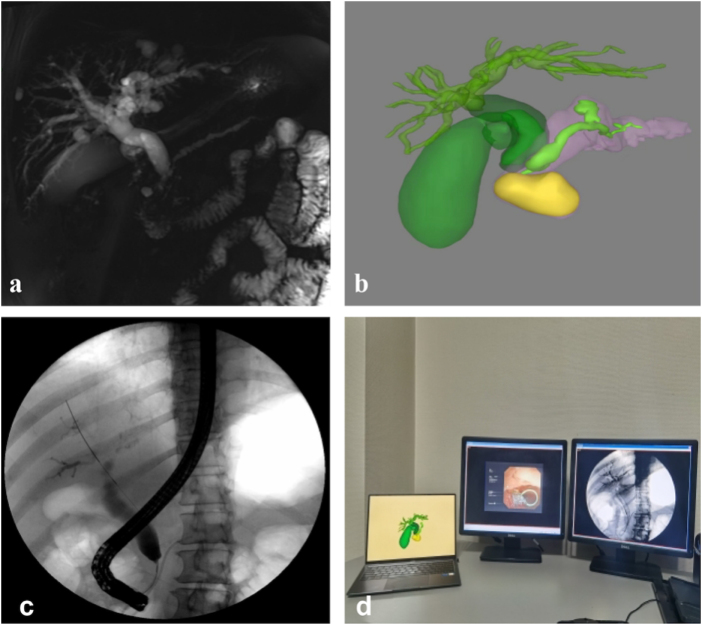
Pancreatic cancer with obstructive jaundice. (a) MRCP image; (b) three-dimensional (3D) reconstruction image; (c) ERCP cholangiogram image; (d) free-rotating 3D images of the biliary tract, endoscopic video, and two-dimensional fluoroscopy images.

In the conventional ERCP group, cholangiopancreatography was performed to assess the location and extent of the bile duct and pancreatic duct strictures, as well as the degree of ductal dilation above the obstruction. Patients received appropriate biliary stents selected and placed based on the stricture location and anatomy. Stent diameter and length were selected according to standard clinical practice. In the 3D-assisted ERCP group, reconstructed 3D images were used for both preoperative planning and intraoperative reference. Endoscopists had access to the endoscopic video, the 3D image of the bile ducts (which could be freely rotated), and the 2D fluoroscopic image simultaneously during the ERCP procedure, as shown in Figure 1. Each center’s cases were managed by a dedicated ERCP team, consisting of a senior endoscopist (with experience in over 750 ERCP procedures) and a fixed assistant. The same team performed procedures across both groups within each center, thereby ensuring operator skill consistency and minimizing interoperator variability.

### Outcome measures

The primary endpoints were the success rate of common bile duct cannulation during ERCP, cannulation time, and X-ray exposure time. The secondary endpoints included clinical success (defined as a ≥50% reduction in serum bilirubin within 5 days), the incidence of procedure-related adverse events within 7 days post procedure (such as pancreatitis, biliary tract infection, gastrointestinal bleeding, and perforation), and the rate of reintervention.

### Statistical analysis

Data analysis was carried out using IBM SPSS version 23.0 software. Continuous variables are presented as means with standard deviations (SDs) or medians with interquartile ranges (IQRs). Categorical variables were presented as frequencies (%). The chi-square test (*χ*^2^) or Fisher’s exact test was used to compare categorical data, while the two-sample *t*-test or the Wilcoxon rank-sum test was used to compare continuous variables. A *P*-value of <0.05 was considered statistically significant.

## Results

### Study patients

A two-center prospective study was conducted, and 72 patients were enrolled between 2021 and 2023. Among them, 36 patients were assigned to the ERCP group, and 36 patients to the 3D-assisted ERCP group. The baseline characteristics are provided in Table 2. All 72 patients had malignant biliary obstruction caused by pancreatic cancer. Additionally, the average baseline total bilirubin levels were 199.3 μmol/l (IQR: 135.5–264.5) in the ERCP group and 213.0 μmol/l (IQR: 158.9–265.1) in the 3D-assisted ERCP group. Overall, there were no statistically significant differences between the two groups in terms of age, sex, pancreatic mass size, common bile duct diameter, the types of stent, or liver function tests (TBiL, DBiL, GGT, ALT, and ALP) before the procedures.

**Table 2:** Baseline characteristics and procedure details

	All (*n* = 72)	ERCP (*n* = 36)	3D-assisted ERCP (*n* = 36)	*P-*value
Age, y (mean ± SD)	62.7 ± 8.6	63.4 ± 8.8	62.1 ± 8.2	0.5071
Gender, n (%)				0.6365
Male	39 (54.2)	18 (50)	21 (58.3)	
Female	33 (45.8)	18 (50)	15 (41.7)	
Size of pancreatic mass, (mm)	31.9 ± 9.1	31.8 ± 9.0	32.0 ± 9.3	0.9495
Common bile duct diameter, (mm)	14.2 ± 3.2	13.8 ± 2.8	14.5 ± 3.5	0.4015
Stent(plastic/metal)	17/55	9/27	8/28	0.7814
TBiL, μmol/l (IQR)	206.1 (139.1–265.1)	199.3 (135.5–264.5)	213.0 (158.9–265.1)	0.4851
GGT, U/l (IQR)	798.1 (443–980)	787.7 (528.3–953.0)	809.4 (421–1126)	0.8541
ALT, U/l (IQR)	213.7 (82.0–301.3)	239.3 (93.3–370.8)	188.1 (81.0–245.5)	0.1489
ALP, U/l (IQR)	477.0 (296.0–545.2)	497.6 (307.3–541.3)	456.3 (268.5–545.3)	0.5989

3D, three-dimensional; ALP, alkaline phosphatase; ALT, alanine aminotransferase; DBiL, direct bilirubin; ERCP, endoscopic retrograde cholangiopancreatography; GGT, γ-glutamyl transpeptidase; IQR, interquartile range;; SD, standard deviation; TBiL, total bilirubin.

### 3D reconstruction outcomes

3D reconstruction was successfully completed for all 36 patients in the 3D-assisted ERCP group (Fig. 1). The reconstructed 3D models provided clear visualization of key anatomical structures, including the location, size, and shape of the pancreatic tumors; the morphology and diameter of the pancreatic and bile ducts; the site and extent of the biliary stricture; the structure of the intrahepatic bile ducts; and their anatomical relationships. The 3D models allowed for 360° rotation, with the ability to add or adjust tissue structures and transparency, enabling precise measurements of the bile and pancreatic ducts’ diameters, as demonstrated in Supplemental Digital Content Video 1, available at http://links.lww.com/JS9/F263. It enables the operating physician to make stereoscopic visual observations of the lesions with omnidirectional, multiple-angle, and multilevel views. Furthermore, the individualized optimal cannulation direction and path suitable for the patients should be accurately selected, and the ERCP operation should be guided preoperatively and intraoperatively.

### ERCP procedure outcomes

In the ERCP group, successful cannulation and stent placement were achieved in 35 of 36 patients. The one patient with failed cannulation underwent successful EUS-guided biliary drainage (EUS-BD). As shown in Table 3, the overall technical success rate in the ERCP group was 97.2% (35/36). In the 3D-assisted ERCP group, all patients achieved successful cannulation and stent placement, yielding a 100% technical success rate. The difference in success rates between groups was not statistically significant. Notably, 3D-assisted ERCP significantly reduced X-ray exposure times for both patients and physicians. The X-ray exposure time was significantly shorter in the 3D-assisted ERCP group compared to the ERCP group (34.5 s [IQR: 24–45.5] vs. 40.5 s [IQR: 28–69], *P* < 0.05), as was the proportion of X-ray exposure (2.66% ± 0.59% vs. 3.11% ± 1.03%, *P* < 0.05). In addition, the mean cannulation time was also significantly shorter in the 3D-assisted ERCP group than in the ERCP group (13.5 min [IQR: 9–21] vs. 16 min [IQR: 13–30], *P* < 0.05). Overall, successful biliary drainage and palliation of cholestasis were achieved in 58 of 72 patients (80.56%), with no significant difference between the two groups (ERCP: 77.78% vs. 3D-assisted ERCP: 83.3%, *P* = 0.77).

**Table 3:** Comparison of outcome measures

	All (*n* = 72)	ERCP (*n* = 36)	3D-assisted ERCP (*n* = 36)	*P-*value
Technical success, *n* (%)	71 (98.6)	35 (97.2)	36 (100)	1
Cannulation time, median (IQR)	15 (10–21)	16 (13–30)	13.5 (9–21)	0.0358^a^
Mean ± SD, min	17.42 ± 9.53	19.78 ± 10.74	15.05 ± 7.41	
Procedure duration, median (IQR)	23 (17–33)	23.5 (17–37)	20.5 (15–31)	0.297
Mean ± SD, min	25.10 ± 11.48	26.53 ± 12.20	23.67 ± 10.51	
X-ray exposure time, median (IQR)	35.5 (25–56)	40.5 (28–69)	34.5 (24–45.5)	0.0337^a^
Mean ± SD, s	43.03 ± 21.94	48.5 ± 24.10	37.53 ± 18.00	
X-ray exposure time to operative time ratio (%)	2.88 ± 0.87	3.11 ± 1.03	2.66 ± 0.59	0.0289^a^
Successful palliation of cholestasis, *n* (%)	58 (80.56)	28 (77.78)	30 (83.3)	0.7668

3D, three-dimensional; ERCP, endoscopic retrograde cholangiopancreatography.

^a^ statistical significance.

### Short-term clinical outcomes

ERCP was successfully performed in 71 of 72 patients (98.6%). After ERCP, symptoms of malignant obstructive jaundice were alleviated or resolved in 58 patients: 28 in the ERCP group (77.78%) and 30 in the 3D-assisted ERCP group (83.3%). At 5 days post procedure, the TBiL in the ERCP group was significantly decreased from 199.3 μmol/l (IQR: 135.5–264.5) to 97.4 μmol/l (IQR: 54.6–118.3) (*P* < 0.05), and the DBiL levels decreased from 128.9 μmol/l (IQR: 81.2–170.4) to 58.3 μmol/l (IQR: 17.4–80.6) (*P* < 0.05). Similarly, in the 3D-assisted ERCP group, TBiL decreased from 213.0 μmol/l (IQR: 158.9–265.1) to 106.6 μmol/l (IQR: 70.7–129.1) (*P* < 0.05), and DBiL dropped from 137.8 μmol/l (IQR: 91.4–174.5) to 69.1 μmol/l (IQR: 40.8–86.4) (*P* < 0.05). ALT and GGT levels were also significantly reduced, and liver function improved after surgery (*P* < 0.05, Table 4). However, there were no significant differences in short-term clinical outcomes between the two groups (*P* = 0.77).

**Table 4:** Preoperative and postoperative laboratory features

	ERCP (*n* = 36)			3D-assisted ERCP (*n* = 36)			*P*-value
Variable	Preoperative	Postoperative	*P–*value	Preoperative	Postoperative	*P–*value	
TBiL (μmol/l)	199.3 (135.5–264.5)	97.4 (54.6–118.3)	<0.0001	213.0 (158.9–265.1)	106.6 (70.7–129.1)	<0.0001	0.5251
DBiL (μmol/l)	128.9 (81.2–170.4)	58.3 (17.4–80.6)	<0.0001	137.8 (91.4–174.5)	69.1 (40.8–86.4)	<0.0001	0.3769
GGT (U/l)	787.7 (528.3–953.0)	454.9 (264–574.3)	0.0001	809.4 (421–1126)	464.5 (255.3–565.3)	0.0047	0.9072
ALT (U/l)	239.3 (93.3–370.8)	104.3 (63.3–143.8)	<0.0001	188.1 (81.0–245.5)	89.4 (49.8–115.3)	<0.0001	0.3165
ALP (U/l)	497.6 (307.3–541.3)	381.2 (209.0–508.0)	0.1146	456.3 (268.5–545.3)	412.8 (220.5–474.8)	0.5398	0.6270

3D, three-dimensional; ALP, alkaline phosphatase; ALT, alanine aminotransferase; DBiL, direct bilirubin; ERCP, endoscopic retrograde cholangiopancreatography; GGT, γ-glutamyl transpeptidase; TBiL, total bilirubin.

### Postoperative complications

The overall rate of procedure-related complications in all patients was 16.67% (12/72), including pancreatitis (7/72, 9.72%), biliary tract infections (4/72, 5.56%), and gastrointestinal bleeding (1/72, 1.39%). There was no significant difference in the complication rates between the two groups, 19.44% (7/36) in the ERCP group and 13.89% (5/36) in the 3D-assisted ERCP group (*P* = 0.75). In addition, there were also no significant differences in the reintervention rates (ERCP: 5.56% vs. 3D-assisted ERCP: 2.78%, *P* = 1) and procedure-related mortality between the two groups (Table 5).

**Table 5:** Post-ERCP complications

	Total (*n* = 72)	ERCP (*n* = 36)	3D-assisted ERCP (*n* = 36)	*P-*value
Procedure-related complication, *n* (%)	12 (16.67)	7 (19.44)	5 (13.89)	0.7531
Pancreatitis	7 (9.72)	4 (11.11)	3 (8.33)	0.9999
Biliary tract infection	4 (5.56)	2 (5.56)	2 (5.56)	1
Gastrointestinal bleeding	1 (1.39)	1(2.78)	0	1
Perforation	0	0	0	1
Requiring reintervention, *n* (%)	3 (4.16)	2 (5.56)	1 (2.78)	1
ERCP	2 (2.78)	1 (2.78)	1 (2.78)	1
Surgical operation	0	0	0	1
Endoscopic hemostasis	1 (1.39)	1 (2.78)	0	1
Procedure-related mortality, *n* (%)	0	0	0	1

3D, three-dimensional; ERCP, endoscopic retrograde cholangiopancreatography.

## Discussion

In recent years, the incidence of pancreatic cancer has increased significantly^[20–22]^. It is estimated that 50% to 80% of patients with pancreatic cancer develop malignant biliary obstruction, commonly presenting as obstructive jaundice. Multiple studies have shown that biliary stent placement, as a nonsurgical and palliative treatment, remains the optimal strategy for relieving malignant obstruction, improving liver function, enhancing quality of life, and prolonging survival^[23,24]^. However, ERCP is still typically performed using 2D fluoroscopic guidance, which exposes patients and medical staff to radiation. Additionally, 2D images from CT scans and cholangiography often fail to clearly depict the spatial structure of the biliopancreatic system, leading to uncertainty in cannulation direction and, at times, blind navigation attempts. With advances in digital medicine, 3D visualization has been increasingly applied in clinical practice. Nevertheless, its application in ERCP for pancreatic cancer patients remains underreported. In this study, we found that 3D reconstruction plays an important role in the successful implementation of ERCP for patients with malignant biliary obstruction caused by pancreatic cancer.

Compared to traditional 2D CT or MR imaging, 3D visualization offers significant advantages in objectively displaying lesions and surrounding anatomical structures^[16]^. It has been widely adopted in fields such as oral and maxillofacial surgery as well as in orthopedics. Previous studies have shown that MRCP is a useful tool for preoperative planning in ERCP. 3D visualization enhances this by clearly showing the anatomical relationship between organs and surrounding structures. Preoperative 3D reconstruction provides a detailed outline of the biliary and pancreatic systems, helping physicians better understand the direction of the biliopancreatic ducts and the positional relationships of pancreatic tumors. The 3D model can be zoomed, rotated, and made semi-transparent, allowing for omnidirectional viewing. This flexibility makes it easier to understand the anatomical features of the biliopancreatic system. Additionally, 3D models can accurately measure the size of pancreatic tumors and the diameter and length of bile and pancreatic ducts, aiding in both preoperative planning and real-time intraoperative navigation. Beyond its immediate clinical utility, we believe our study contributes meaningful translational value by establishing a feasible and effective 3D-assisted ERCP workflow in a real-world setting.

Before surgery, 3D reconstruction technology enables doctors to accurately locate and map the biliary and pancreatic ducts, facilitating optimal procedural planning and minimizing blind exploration during the procedure^[25]^. Intraoperatively, 3D reconstruction can assist in navigating the ERCP procedure, aiding in selective cannulation and guiding the selection and placement of biliary stents. By improving spatial orientation, 3D guidance contributes to shorter procedural durations, enhanced precision, and lower operative risk. In our study, the use of 3D assistance significantly reduced both cannulation time and X-ray exposure, demonstrating that the application of 3D reconstruction in ERCP can improve procedural safety. Although the 3D-assisted group demonstrated a higher clinical success rate and a lower incidence of procedure-related adverse events, these differences did not reach statistical significance, possibly due to the limited sample size. Furthermore, the short follow-up period may not have been sufficient to capture delayed complications or assess long-term clinical benefits. Future studies with larger sample sizes and extended follow-ups are warranted to further evaluate these outcomes.

Contrast agents are often required to outline strictures during ERCP, posing a potential risk of radiation exposure to both medical staff and patients^[13,14]^. The American Society for Gastrointestinal Endoscopy (ASGE) recommends routine monitoring of fluoroscopy time and radiation dose as quality benchmarks for ERCP^[13,15]^. Additionally, contrast injection into intrahepatic ducts in the setting of distal biliary obstruction may predispose patients to cholangitis. Our results demonstrate that 3D reconstruction significantly reduces X-ray exposure time and the use of contrast media. However, no significant difference in the incidence of cholangitis was observed between the two groups. Further larger, controlled studies are needed to validate this finding.

Previous studies have reported that the success rate of ERCP is closely related to the operator’s experience and their ability to interpret images^[26]^. A recent multicenter study evaluating trainee competence found that after a 1-year advanced endoscopy fellowship, the overall technical competence for ERCP was only 60%^[27]^. This remains considerably lower than the success rates typically achieved by experienced endoscopists. Notably, 3D reconstruction technology has been shown to enhance immersion and accessibility in surgical training. For example, its use has improved teaching effectiveness in oncological colorectal surgery among young surgeons^[28]^. A prospective study demonstrated that ERCP training using a silicone simulator created with 3D printing technology significantly improved trainees’ ERCP techniques^[29]^. Based on these findings, we believe that incorporating 3D reconstruction into ERCP training programs may help shorten the learning curve and improve procedural confidence among less experienced endoscopists. This technology offers clearer and more detailed visualization of the biliary and pancreatic anatomy, which can enhance both procedural planning and intraoperative navigation.

While 3D reconstruction shows promise in improving the safety and efficiency of ERCP, its routine implementation in clinical practice may involve additional cost and resource requirements. These include the need for advanced imaging workstations, trained personnel for image processing, and additional time for 3D reconstruction. Therefore, future cost-effectiveness analyses are warranted to evaluate the economic feasibility and long-term value of integrating 3D reconstruction in routine ERCP workflows, particularly in high-volume centers.

There were several study limitations. First, the small sample size may have limited the statistical power to detect significant differences. Second, the short follow-up period prevented the assessment of long-term outcomes. Third, to minimize clinical heterogeneity and focus on the value of 3D reconstruction in anatomically complex cases, we included only patients with malignant biliary obstruction secondary to pancreatic cancer. It may introduce selection bias and limit the generalizability of our findings. Future large-scale, multicenter studies with broader disease inclusion and longer follow-ups are warranted to validate and extend our results.

## Conclusion

In conclusion, 3D reconstruction-assisted ERCP significantly reduces cannulation time and X-ray exposure time in the treatment of malignant biliary obstruction secondary to pancreatic cancer, thereby enhancing procedural safety and efficiency. This technology shows considerable promise for improving clinical outcomes and procedural success in therapeutic endoscopy, particularly in complex ERCP cases. However, this study has limitations, including a small sample size, limited generalizability, and lack of long-term follow-ups. Further large-scale studies are warranted to confirm these results and evaluate long-term benefits.

## Data Availability

The raw data generated and analyzed in the current study are not publicly available due to appropriate protection of patient personal information but are available from the corresponding author upon reasonable request.

## Research registration unique identifying number (UIN)

ChiCTR2100042469.
